# DNA Damage Induced MutS Homologue hMSH4 Acetylation

**DOI:** 10.3390/ijms141020966

**Published:** 2013-10-18

**Authors:** Yen-Lin Chu, Xiling Wu, Jing Xu, Jennifer L. Watts, Chengtao Her

**Affiliations:** School of Molecular Biosciences, Mail Drop 64-7520, Washington State University, Pullman, WA 99164-7520, USA; E-Mails: y1chu@ucsd.edu (Y.-L.C.); xilingwu@vetmed.wsu.edu (X.W.); jingxu@vetmed.wsu.edu (J.X.); jwatts@vetmed.wsu.edu (J.L.W.)

**Keywords:** hMSH4, hMof, acetylation, HDAC3, DSB, ionizing radiation, NHEJ

## Abstract

Acetylation of non-histone proteins is increasingly recognized as an important post-translational modification for controlling the actions of various cellular processes including DNA repair and damage response. Here, we report that the human MutS homologue hMSH4 undergoes acetylation following DNA damage induced by ionizing radiation (IR). To determine which acetyltransferases are responsible for hMSH4 acetylation in response to DNA damage, potential interactions of hMSH4 with hTip60, hGCN5, and hMof were analyzed. The results of these experiments indicate that only hMof interacts with hMSH4 in a DNA damage-dependent manner. Intriguingly, the interplay between hMSH4 and hMof manipulates the outcomes of nonhomologous end joining (NHEJ)-mediated DNA double strand break (DSB) repair and thereby controls cell survival in response to IR. This study also shows that hMSH4 interacts with HDAC3, by which HDAC3 negatively regulates the levels of hMSH4 acetylation. Interestingly, elevated levels of HDAC3 correlate with increased NHEJ-mediated DSB repair, suggesting that hMSH4 acetylation *per se* may not directly affect the role of hMSH4 in DSB repair.

## 1. Introduction

Protein acetylation was originally recognized as an important post-translational modification of histones during transcription and DNA repair [1]. Recently, however, the arena of acetylation has been extended to include non-histone proteins, particularly those involved in the process of DNA double strand break (DSB) repair [2–5]. In fact, it has been recently demonstrated that acetylation regulates the key DNA damage response kinases ATM and DNA-PKcs [2,4], as well as a plethora of DNA repair factors including NBS1, Ku70, and p53 [3,6–9]. These evidences tend to support a pivotal role for acetylation in the process of DNA damage response and repair—ostensibly through facilitating the recognition and signaling of DNA lesions, as well as orchestrating protein interactions to recruit activities needed in the process of the repair. Specifically, acetylation is critical in the activation of DNA damage response pathways [2,4]. In spite of these advances, precise functional roles of acetylation of the most non-histone DNA repair proteins are still elusive. Recent research suggests that this covalent protein post-translational modification could also confer new functional properties, and thus modified proteins can carry out distinct roles. Indeed, it has been well documented that Ku70 and p53 acetylation are involved in promoting apoptosis [6,8,10]. While p53 and Ku70 interaction is acetylation-independent, p53 acetylation facilitates the dissociation of BAX from Ku70 and therefore enhances apoptosis [7]. Due to these observations, it is presently believed that non-histone acetylation is widely spread and modulates a multitude of protein functions [2].

This widespread pattern of protein acetylation is conceivably maintained through the action of many lysine acetyltransferases. To date, the known acetyltransferases can be classified into three families (*i.e.*, Gcn5/PCAF, p300/CBP, and MYST) on the basis of their amino acid sequence similarity [5]. Over the past several years, an increasing number of lysine acetyltransferases have been implicated in the process of DNA damage response and repair mainly through modification of non-histone proteins. For example, p300/CBP and PCAF are involved in mediating DNA damage response [6]. Likewise, the MYST acetyltransferases Tip60 (*i.e*., 60 kDa Tat-interactive protein) and hMof (*i.e*., males absent on the first) participate directly in DNA damage repair through controlling the functions of ATM, DNA-PKcs, p53, and c-Abl [11–14].

Although there is ample evidence underscoring the necessity of acetylation in DSB repair, the extent of protein acetylation in DNA damage repair is still unclear. In this study, we demonstrate that the human MutS homologue hMSH4 undergoes DNA damage-induced acetylation. Despite the fact that hMSH4 is a member of the MutS protein family [15], to date there is no evidence for its participation in conventional mismatch repair MMR [16]. Cumulated evidence, however, has suggested a role for hMSH4 in meiotic recombinational DSB repair [16–19]. In *C. elegans*, silencing of BRCA1 orthologue on a MSH4-deficient background leads to chromosome fragmentation during meiosis [20], indicating a potential synergistic effect between hMSH4 and BRCA1 on DSB processing. It is known that hMSH4 interacts with an array of protein factors—which currently include hMSH5, hMLH1, hMLH3, hRad51, DMC1, GPS2, VBP1, and eIF3f—associated with diverse cellular functions [16,21–29]. This hMSH4 protein interaction profile is not only compatible with a role of hMSH4 in DSB repair, but also supports the idea that hMSH4 may exert multiple functions through interacting with different protein partners. In the present study, we have investigated DNA damage-induced hMSH4 acetylation and deacetylation, and have identified new hMSH4-interacting proteins that are responsible for these post-translational modifications and their roles in NHEJ-mediated DSB repair.

## 2. Results

### 2.1. hMSH4 Is Acetylated in Response to DNA Damage

**Figure 1:**
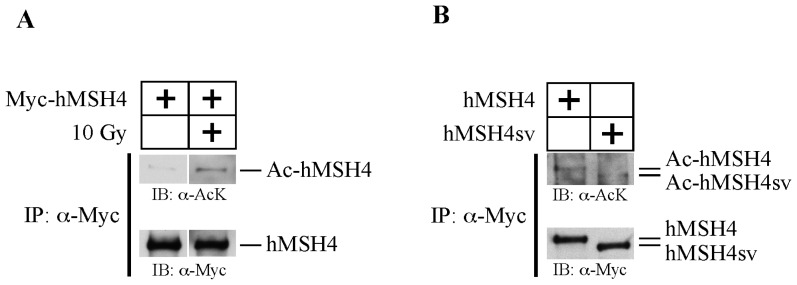
DNA damage induces hMSH4 acetylation. (**A**) Analysis of hMSH4 acetylation in response to IR-induced DNA damage. 293T cells expressing full-length hMSH4 were irradiated by 10 Gy IR. The levels of hMSH4 acetylation were analyzed 6 h after IR treatment by immunoblotting of immunopurified hMSH4 protein performed with the α-Acetylated-Lysine antibody (α-AcK); (**B**) Analysis of the basal level of hMSH4 acetylation. Full-length hMSH4 and hMSH4sv were separately expressed in 293T cells and purified by immunoprecipitation. The levels of acetylation were analyzed by immunoblotting.

It has been increasingly recognized that protein acetylation plays important roles in the process of DSB repair [2], but the possible involvement of acetylation in modulating proteins of the MMR family remains unexplored. The human MMR family member hMSH4 is a MutS homologue protein previously implicated in the process of DSB repair that likely depends on the formation of a heterocomplex with hMSH5 [18,30]. In the present study we first tested the possibility that hMSH4 might be post-translationally modified by acetylation in human cells. To this end, 293T cells were transfected to express Myc-tagged hMSH4 and were treated with 10 Gy ionizing radiation (IR) at 48 h post transfection. The α-Myc antibody was used to perform immunoaffinity purification of hMSH4 proteins from the control and IR-treated cells. Immunoblotting analysis of purified hMSH4 protein indicated that IR-induced DNA damage elevated the levels of hMSH4 acetylation significantly above the basal level of acetylation (Figure 1A).

To further validate the basal hMSH4 acetylation, Myc-tagged hMSH4 and hMSH4sv (*i.e*., splicing variant truncated at the carboxyl terminal) [25] were expressed in 293T cells and immunoaffinity-purified hMSH4 and hMSH4sv were both positively reactive with the α-Acetylated-Lysine antibody (Figure 1B). These findings indicate that hMSH4 is modified by acetylation, and the altered *C*-terminus of hMSH4 does not affect this modification. Together, the evidence indicates that hMSH4 is acetylated in human cells and that DSB-inducing agents can promote hMSH4 acetylation.

### 2.2. hMSH4 Physically Interacts with hMof

**Figure 2:**
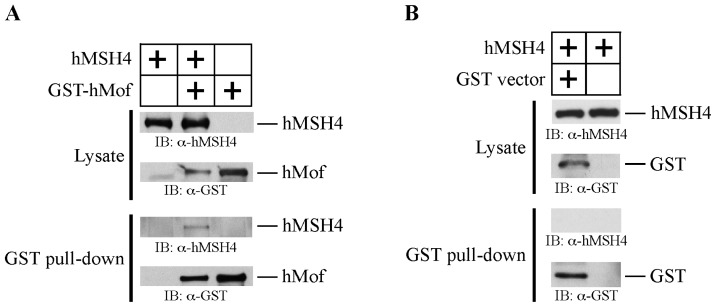

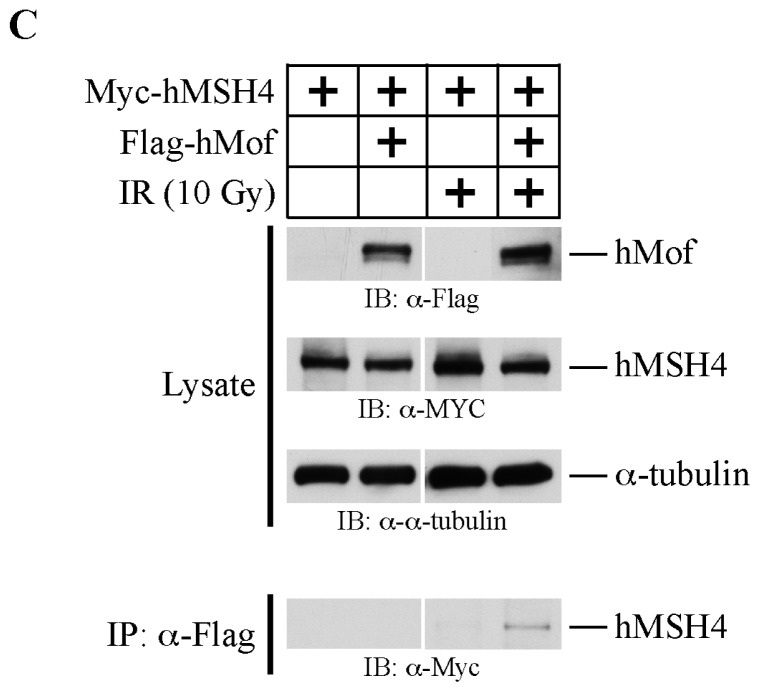
hMSH4 interacts with hMof. (**A**) Recombinant hMof was produced as a glutathione *S*-transferase-tagged fusion protein and was co-expressed with hMSH4. Soluble cell lysates were used for GST pull-down analysis. Western blot analysis was performed to detect the expression of hMSH4 protein; (B) Negative controls for GST pull-down assay. In the absence of GST-hMof, glutathione-Sepharose 4B beads could not directly pull down hMSH4 even in the presence of GST tag; (**C**) Co-immunoprecipitation analysis of hMSH4 and hMof interaction in human cells. Myc-hMSH4 and Flag-hMof expression in 293T cells was validated by Western blotting. IR treatment was performed 48 h after transfection. The α-Flag antibody was used to perform co-immunoprecipitation analysis, and co-immunoprecipitated hMSH4 was validated by Western blot analysis.

The observation that hMSH4 acetylation could be elevated in cells possessing increased levels of DSBs raised the possibility that hMSH4 may be modified by one or more of the acetyltransferases involved in DNA damage response. To test this possibility, GST pull-down analysis was performed using bacterially expressed proteins to determine potential interactions of hMSH4 with hMof, hGCN5, and hTip60. Fusion His_6_-hMSH4 or GST-hMSH4 protein was co-expressed with one of the three acetyltransferases, and each of these proteins was also expressed individually in BL21 (DE3)-RIL cells as controls. We found that hMSH4 could be co-purified with GST-hMof by glutathione-Sepharose 4B beads, and hMSH4 pull-down was completely dependent on the expression of hMof (Figure 2A). In order to ensure that GST protein alone or glutathione-Sepharose 4B beads could not directly pull down hMSH4, GST pull-down analysis was performed with cell extracts containing either hMSH4 alone or hMSH4 and GST protein. The results demonstrated that neither GST tag nor glutathione-Sepharose 4B beads were able to pull-down hMSH4 (Figure 2B). Furthermore, GST pull-down experiments demonstrated that hMSH4 also interacted with hGCN5 (data not shown). However, similar experiments illustrated that hMSH4 could not interact with hTip60.

### 2.3. The hMSH4-hMof Interaction Is IR-Inducible in Human Cells

To test whether hMSH4 could interact with hMof or hGCN5 in human cells, 293T cells were transfected to express Myc-hMSH4 and Flag-hMof or Flag-hGCN5. One set of transfected cells was irradiated with 10 Gy IR at 48 h post transfection. Cell extracts were prepared 6 h post IR treatment. Potential protein interactions between hMSH4 and hMof or hGCN5 were tested by co-immunoprecipitation performed with the anti-Flag antibody. The results presented in Figure 2C clearly indicate that hMSH4 interacts with hMof in IR-treated cells, suggesting that hMSH4 interacts with hMof in a DNA damage-dependent manner. Due to the fact that hMof has a similar molecular weight to that of immunoglobulin heavy chains, reciprocal co-immunoprecipitation is thus not technically feasible. On the other hand, similar experiments performed with hGCN5 in 293T cells yielded no evidence for protein interaction between hMSH4 and hGCN5 (data not shown). Because of this, we have focused on the hMSH4-hMof interaction in all subsequent analyses, although at present we cannot exclude the possibility that only transient or lower than detectable hMSH4-hGCN5 interaction may exist in human cells.

The observed IR-inducible hMSH4-hMof interaction in 293T cells suggests that the physical interaction between these two proteins and the subsequent post-translational modification of hMSH4 are intimately involved in the process of IR-induced DNA damage response. Because bacterially expressed hMSH4 and hMof readily interact with one another (Figure 2A), it is possible that the interaction between hMSH4 and hMof in human cells are tightly regulated, presumably by other protein factors or post-translational modifications. Nevertheless, how cellular signaling from IR-induced DNA damage directs hMSH4 acetylation is presently unknown.

### 2.4. hMof Is Capable of Mediating hMSH4 Acetylation *In Vitro*

**Figure 3:**
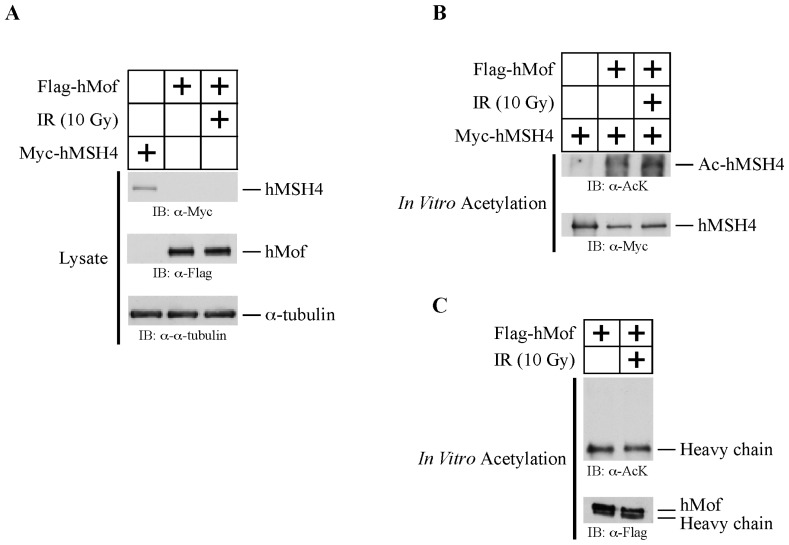
hMof mediates hMSH4 acetylation *in vitro*. (**A**) Western blot analysis of hMSH4 and hMof expression in 293T cells. Cell extracts were prepared 48 h after transfection; (**B**) *In vitro* acetylation analysis (see Materials and Methods for details). Immunoaffinity purified hMSH4 and hMof from IR-treated and control cells were incubated in the *in vitro* acetylation reaction buffer for 15 min, and samples were analyzed by immunoblotting; (**C**) Western blot analysis of immunoaffinity purified hMof. When the *in vitro* acetylation assay was performed with hMof alone, there was no detectable lysine acetylation signal within the range of molecular weights similar to that of hMSH4. This blot served as a specificity control for the *in vitro* acetylation assay.

To further confirm that hMof was responsible for the acetylation of hMSH4, we performed *in vitro* acetylation analysis of hMSH4 and hMof (see Materials and Methods for details). In this experiment, hMSH4 and hMof were individually expressed in 293T cells, and one set of cells expressing hMof was irradiated with 10 Gy IR at 48 h post transfection. Because IR treatment is known to activate hMof-dependent acetylation of histone H4 and ATM activation [11], we hypothesized that IR could trigger hMof activation and in turn facilitate hMSH4 acetylation. The expression of individual proteins was validated by Western blotting analysis (Figure 3A). Expressed hMSH4 and hMof proteins were individually purified by immunoprecipitation with α-Myc and α-Flag antibodies and were used to perform the *in vitro* acetylation assay (Figure 3B). The results of the *in vitro* acetylation analysis indicated that incubation with immunoaffinity-purified hMof resulted in hMSH4 acetylation (Figure 3B). In particular, it appeared that hMof from IR-treated cells could slightly enhance hMSH4 acetylation (Figure 3B). Given the observation that IR could induce hMSH4-hMof interaction and hMSH4 acetylation (Figures 1C and 2C), the lack of an obvious IR-dependent enhancement of *in vitro* hMSH4 acetylation most likely suggests that the interplay between hMSH4 and hMof is subjected to additional regulation *in vivo*, and it is negatively regulated under normal physiological conditions. Collectively, the *in vitro* acetylation analysis clearly demonstrates that hMSH4 is an hMof substrate.

### 2.5. hMof Modulates the Effect of hMSH4 on NHEJ-Mediated DSB Repair and Cell Survival to IR

**Figure 4:**
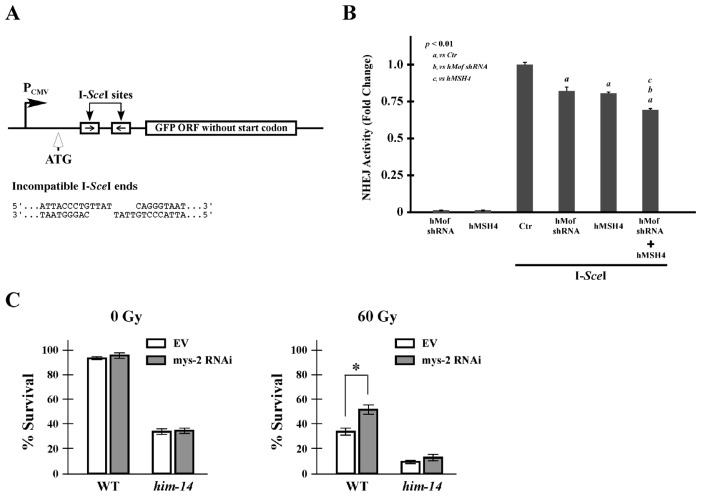
hMof modulates the effect of hMSH4 on NHEJ-mediated DSB repair and cell survival in response to IR. (**A**) Schematic representation of the NHEJ reporter locus. The relative locations of the ATG start codon, the I-*Sce*I recognition sites, and the CMV promoter (**P**_CMV_) are indicated; (**B**) Analysis of the effects of hMof and hMSH4 on NHEJ. Expression constructs encoding I-*Sce*I, hMof sh-2, and hMSH4 were transfected into the NHEJ reporter cell line 293T/#8-1 as indicated. The hMof knockdown construct, hMof sh-2, was found to be able to silence approximately 90% of hMof protein expression (data not shown). Cells were analyzed by FACS at 48 h post-transfection. Average NHEJ activities of three measurements were graphed. Error bars are standard deviation of the mean; (**C**) Depletion of *mys-2* protects wild type *C. elegans* from IR exposure. Graphs show the survival rate of embryos laid by wild type (N2) and *him-14* hermaphrodites exposed to 0 or 60 Gy of IR. Data are the average of at least 5 replicates from two radiation exposures (* *p* < 0.05).

Since hMSH4 is known to suppress NHEJ-mediated DSB repair [29], we next tested whether hMof exerted a similar effect on the process. Specifically, the 293T/#8-1 NHEJ reporter cell line was utilized to assess the effect of hMof knockdown on NHEJ-mediated DSB repair (Figure 4A). To perform this analysis, pCBA-(I-*Sce*I) was transfected into the 293T/#8-1 NHEJ reporter cell line together with hMof RNAi and/or hMSH4 expression constructs. The results of these experiments indicated that RNAi-mediated hMof silencing compromised NHEJ to a level comparable to that mediated by hMSH4 overexpression (Figure 4B). Interestingly, hMof silencing in the hMSH4 overexpression background further decreased NHEJ activity (Figure 4B), suggesting that hMof can antagonize the suppressive effect of hMSH4 on the mutagenic NHEJ-mediated DSB repair.

To test for a physiological interaction between MOF and MSH4 in the context of a whole organism, we used *C. elegans* to examine the effect of depletion of *mys-2* (the *C. elegans* MOF homolog) in the wild type and *him-14* (MSH4 homolog) mutant strains [31,32]. Embryo survival in *C. elegans* is a sensitive measure of erroneous DSB repair and chromosomal instability. HIM-14/MSH4 plays an important role in the maintenance of chromosomal stability by promoting faithful HR-mediated repair of DSBs in *C. elegans* [32]. Consistent with this, HIM-14/MSH4 deficiency impairs *C. elegans* embryo survival (Figure 4C). In addition, we found that *mys-2* RNAi treatment had no effect on embryo survival in either worm strain grown under laboratory conditions (Figure 4C). However, after exposure to IR, embryos laid by exposed wild type worms that were treated with *mys-2* RNAi showed an increased hatching and survival rate compared to those treated with the empty control vector (Figure 4C). Together with the NHEJ reporter analysis, these observations indicate that MYS-2/MOF antagonizes the suppressive effect of HIM-14/MSH4 on erroneous DSB repair, and the effect of *mys-2* RNAi requires functional HIM-14/MSH4 (Figure 4C).

### 2.6. hMSH4 Interacts with Histone Deacetylase 3 (HDAC3)

The existence of low basal levels of hMSH4 acetylation suggests that hMSH4 acetylation might be actively monitored in human cells. We have previously demonstrated that the interface of hMSH4-hMSH5 complex interacts with GPS2 [27], which is an integral component of the HDAC3 complex [33]. It is also noteworthy that both HDAC3 and hMof act on histone H4 during DSB repair [11,34]. Together, it is plausible that HDAC3 may act on acetylated hMSH4. Hence, we examined the interaction between HDAC3 and hMSH4-hMSH5 by yeast three-hybrid analysis (Table 1).

**Figure 5:**
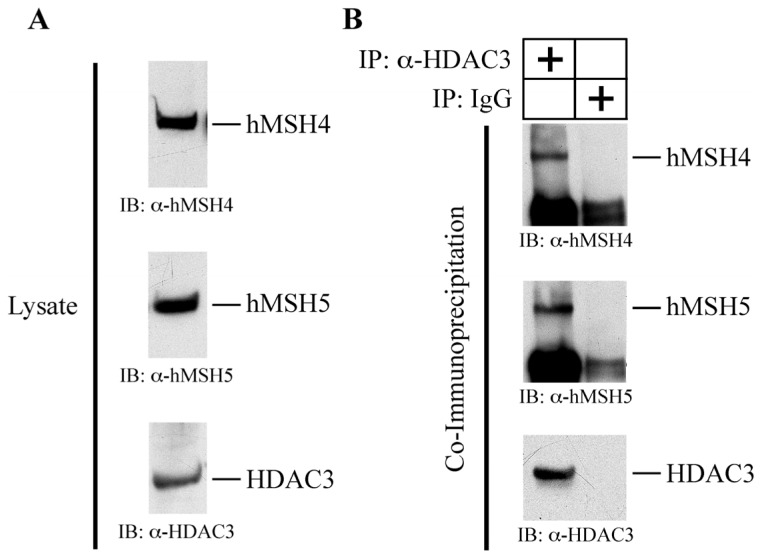
Co-existence of hMSH4 and HDAC3 in the same protein complex in human cells. (**A**) Western blotting analysis of relevant protein expressions in 293T/f45 cells; (**B**) Co-immunoprecipitation analysis of HDAC3 interaction with hMSH4 and hMSH5. Anti-HDAC3 antibodies were used to immunoprecipitate endogenous HDAC3, and the presence of hMSH4 and hMSH5 in the immunoprecipitates were detected by Western blotting with the α-hMSH4 and α-hMSH5 antibodies.

Consistent with previous studies, three-hybrid analysis showed that GPS2 interacted with the hMSH4-hMSH5 heterocomplex (Table 1). While HDAC3 interacted with neither hMSH4 nor hMSH5 alone, three-hybrid analysis demonstrated that HDAC3 interacted with the hMSH4-hMSH5 heterocomplex (Table 1). However, the positive interaction was only observed with the AD-HDAC3, BD-hMSH4, and HA-hMSH5 configuration, suggesting that the interaction with AD-HDAC3 is conformation sensitive. This observation also indicates that hMSH5-binding could facilitate hMSH4 to adopt a suitable configuration for HDAC3 interaction. It should be noted that both of the amino and carboxyl terminal regions of hMSH5 are required to form a composite domain for hMSH4-hMSH5 interaction, whereas this interaction only involves with the carboxyl terminal end of hMSH4 [27]. To further validate the interaction between HDAC3 and hMSH4-hMSH5 in human cells, co-immunoprecipitation analysis was performed using 293T/f45 cells [27]. As shown in Figure 5, α-HDAC3 (rabbit polyclonal) co-immunoprecipitated hMSH4 and hMSH5 from 293T/f45 cell extracts, suggesting that HDAC3 coexisted in the same complex with hMSH4-hMSH5 in human cells. Furthermore, the co-immunoprecipitation experiments with 293T cells expressing hMSH4 or hMSH4sv demonstrated that HDAC3 interacted with both the full-length hMSH4 and hMSH4sv (data not shown). Although the exact mechanism of HDAC3 association with hMSH4 and/or hMSH5 in human cells remains to be delineated, the co-existence of these proteins in the same complex suggests that HDAC3 is likely involved in controlling the levels of hMSH4 acetylation.

### 2.7. HDAC3 Facilitates hMSH4 Deacetylation

**Figure 6:**
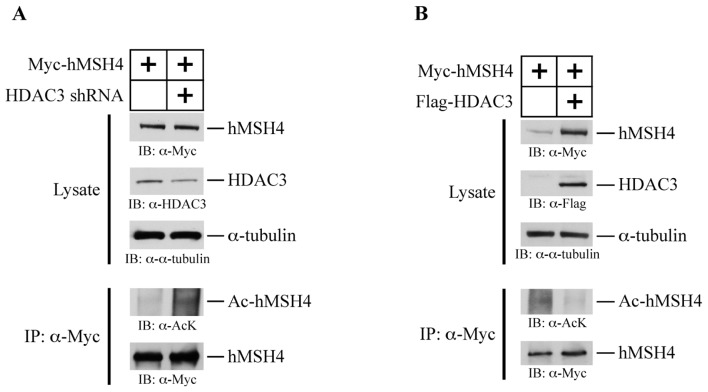
Effects of HDAC3 RNAi and HDAC3 over-expression on hMSH4 acetylation. (**A**) Effects of HDAC3 RNAi on hMSH4 acetylation. HDAC3 knockdown was achieved by transient transfection of 293T cells with the HDAC3 shRNA-encoding construct and validated with immunoblotting with α-HDAC3 antibody. The levels of hMSH4 acetylation under different conditions were measured by immunoblotting performed with the α-Acetylated-Lysine antibody; (**B**) Effects of HDAC expression on hMSH4 acetylation. Over-expression of HDAC3 in 293T cells was carried out by transient transfection, and the levels of over-expression were validated by Western blot analysis performed with α-Flag antibody. Corresponding levels of hMSH4 acetylation were determined by immunoblotting.

The observed low basal levels of hMSH4 acetylation are highly suggestive of a mechanism that tightly controls hMSH4 acetylation. In order to test whether HDAC3 played a role in controlling the status of hMSH4 acetylation, the effects of RNAi-mediated HDAC3-silencing as well as over-expression of HDAC3 on hMSH4 acetylation were investigated. Specifically, RNAi-mediated HDAC3-silencing was performed in conjunction with hMSH4 expression in 293T cells. Transfection of 293T cells with an shRNA encoding construct pmH1P-neo/HDAC3 sh-1 led to an approximately 50% reduction of HDAC3 expression (Figure 6A). Western blot analysis of equivalent amounts of immunoaffinity-purified hMSH4 from 293T cells and HDAC3-silenced counterparts showed that hMSH4 was subjected to HDAC3-mediated deacetylation (Figure 6A). To further confirm that HDAC3 was responsible to deacetylate hMSH4, the effects of HDAC3 over-expression on hMSH4 acetylation was also examined in 293T cells. Western blot analysis of similar amounts of immunoprecipitated hMSH4 protein indicated that over-expression of HDAC3 resulted in a reduced level of hMSH4 acetylation (Figure 6B). These observations clearly demonstrate that HDAC3 is involved in the process of hMSH4 deacetylation.

## 3. Discussion

It has been recently recognized that lysine residues of non-histone proteins—involved in many different biological processes including DNA damage recognition and repair—are frequently acetylated in a reversible fashion. In fact, most protein acetylation is controlled by both histone acetyltransferases (HATs) and HDACs; therefore, the levels of acetylation can be quickly adjusted to tailor protein functions in response to cellular requirements. Our current study demonstrates that hMSH4 becomes acetylated in response to IR-induced DNA damage. This DNA damage-triggered hMSH4 acetylation is mediated by hMof—one of the well-known DNA damage response acetyltransferases [35]. The tissue expression profiles of hMSH4 and the MYST family acetyltransferases, *i.e*. hTip60 and hMof, are very similar [36], which supports the idea that the interplay of these proteins could exist in a variety of cell types. In addition, our study has also demonstrated that hMSH4 can be deacetylated by HDAC3. Collectively, our data indicate that hMSH4 acetylation is dynamically regulated by hMof and HDAC3. Consistent with observations implicating hMSH4 in the HR process, both hMof and HDAC3 are known to play important roles in the process of DSB repair [11,34]. This supports a scenario in which both acetylation and deacetylation attribute to the function of hMSH4 in DSB repair.

The results of our present study also suggest that hMof antagonizes the suppressive effect of hMSH4 on the mutagenic NHEJ-mediated DSB repair. In conjunction with the known protein interaction profile of hMSH4 with HR proteins [16], hMSH4 acetylation could likely serve as a mechanism to regulate protein-protein interaction during DNA damage recognition and repair. Given the constitutively low levels of hMSH4 expression in human cells [15,25], acetylation might temporally change hMSH4 protein stability and/or conformation, presumably through the competition with lysine polyubiquitination—a modification known to mediate hMSH4 degradation [37]. Furthermore, the timing of hMSH4 acetylation in response to DNA damage may be also pertinent to the role of hMSH4 in the repair process.

Several studies have linked hMSH4 to disease conditions in humans. A recently study reported that *hMSH4* expression in the breast cancer cell line MCF-7 was down-regulated due to DNA hypermethylation [38]. The *hMSH4* non-synonymous SNP G^289^→A (*i.e*., encoding hMSH4^Ala97Thr^) has been associated with an increased risk for breast cancer [39], while *hMSH4* G^1243^→A (*i.e*., encoding hMSH4^Glu415Lys^) has been identified as an important marker for blood malignancy [40]. Studies in *C. elegans* have previously shown that the orthologues of hMSH4 and BRCA1 acted synergistically in the maintenance of chromosome stability [20]. In addition, loss of chromosomal region 1p31-32, harboring *hMSH4* and several other genes, in myeloma patients is significantly associated with shorter survival [41]. These observations have underscored the possibility that hMSH4 is important for the maintenance of chromosome stability even though it is normally expressed at a very low level.

Since the hMSH4 and hMof interaction in human cells occurs only after the induction of DNA damage, the basal level of hMSH4 acetylation is likely to be maintained by acetyltransferases through transient interactions. It is plausible that, in addition to hMof, hGCN5 may potentially contribute, at least to certain extent, to the basal hMSH4 acetylation. Although the role of induced hMSH4 acetylation in DNA damage response still remains to be defined, the results of our current study have also raised several other interesting possibilities. First and foremost, this DNA damage-induced hMSH4 acetylation might play a role in the regulation of protein-protein interactions. Thus, it would be critical to determine whether hMSH4 acetylation poses any effects on its interaction with hMSH5—an altered hMSH4-hMSH5 interaction can potentially exert a significant impact on the interplay of hMSH5 with c-Abl in DNA damage response and repair [30,42,43]. This is also pertinent to the catalytic outputs of c-Abl in regulating the balance between DSB repair and the activation of cell death response [42,44,45]. Finally, the nuclear functions of hMSH4 and its interacting partner hMSH5 are likely harnessed by mechanisms governing nuclear-cytoplasmic protein trafficking [46]. Therefore, it would be interesting to know whether hMSH4 acetylation may have any effect on nuclear-cytoplasmic protein redistribution. Answers to these questions will certainly lead to new avenues for future studies of the biological functions of hMSH4 in DSB damage response and repair processes.

## 4. Experimental Section

### 4.1. Cell Culture, Cell Extracts, and Induction of DNA Damage

HeLa and 293T-derived cell lines were maintained in DMEM (Invitrogen, Carlsbad, CA, USA) containing 10% FBS (Atlanta Biologicals, Lawrenceville, GA, USA) and 1× Antibiotic-Antimycotic (Invitrogen). 293T/f45 is a previously generated stable cell line expressing both hMSH4 and hMSH5 [27]. Whole cell extracts were prepared by the use of the CelLytic M Cell Lysis Reagent (Sigma, St. Louis, MO, USA) containing 1× Halt Protease Inhibitor Cocktail (Thermo Scientific, Rockford, IL, USA). Irradiation was performed at room temperature with a cobalt-60 source at a dose rate of 6.6 or 4.45 Gy/min (Nuclear Radiation Center, Washington State University, WA, USA).

### 4.2. SDS-PAGE, Western Blotting, Co-Immunoprecipitation (Co-IP) and Antibodies

Cell extracts and immunoprecipitates were separated by 4%–20% gradient SDS-PAGE and were transferred to nitrocellulose membranes (Bio-Rad Laboratories, Hercules, CA, USA). Subsequent immunoblotting analyses were performed with the Pierce ECL Western Blotting Substrate (Thermo Scientific, Rockford, IL, USA). Five to 10 μg of relevant antibodies were used to perform immunoprecipitation analysis. Immunoprecipitates were captured with 40 μL of 50% slurry of BSA-saturated Protein A/rProtein G-Agarose beads (Invitrogen). Antibodies used in the present study include α-Myc (Clontech, Mountain View, CA, USA), α-hMSH4 [25], α-hMSH5 [42], α-FLAG M2 (Sigma), α-α-tubulin (Sigma), α-HDAC3 (mouse monoclonal 3G6) (Upstate Laboratories, East Syracuse, NY, USA), α-HDAC3 (rabbit polyclonal) (Upstate Laboratories), α-HDAC3 (rabbit polyconal, Abcam, Cambridge, MA, USA), α-Acetylated-Lysine (Cell Signaling Technology, Danvers, MA, USA), α-GST (GE Healthcare Life Sciences, Piscataway, NJ, USA) and purified goat IgG (Sigma). Secondary antibodies used in this study include GAM-HRP, GAR-HRP, and DAG-HRP (Bio-Rad Laboratories).

### 4.3. Expression Constructs and Mammalian Transfection

The coding sequences of the full-length hMSH4 and the splicing variant hMSH4sv [25] were cloned into mammalian expression vector pCMV-Myc (Clontech). The expression of full-length hMSH4 and hMSH4sv proteins in 293T cells was carried out by transient transfection with the standard calcium-phosphate procedure. The coding sequences of hMof and hGCN5 were PCR amplified from a human testis cDNA preparation (Clontech). Specifically, the hMof coding sequence was amplified by the use of primers hMof F-6*Kpn* (5′-GGGGTACCCCCGCGATGGCGGCACAGGG AGCT-3′) and hMof R1389*Bam*H (5′-CGCGGATCCGGGCCAGGCTGCTCACTTCTTGGA-3′) and cloned into pPuro-Flag [27]. Likewise, the hGCN5 coding sequence was amplified by the use of primers hGCN5 F1081*Kpn* (5′-GGGGTACCTCCATGCTGGAGGAGGAGATCTAT-3′) and hGCN5 R2532*Eco*R (5′-CGGAATTCTGCGGCCCAAAGATGGGCCTACTT-3′) and subsequently cloned into pPuro-Flag. The mammalian expression construct for hTip60 (pcDNA3.1-N-HA-hTip60) was generously provided by Thomas C. Sudhof [47]. The coding sequence of hTip60 was then subcloned into pPuro-Flag. Flag-tagged HDAC3 was produced from expression construct pEV-589, which was kindly obtained from Eric Verdin [48]. The hMof and HDAC3 silencing shRNA-encoding constructs, pmH1P-neo/hMof sh-2 and pmH1P-neo/HDAC3 sh-1, were constructed as previously described [49], which target hMof or HDAC3 transcript at coding position 427-447 (5′-AAGCGCAAGCATGATGA GATC-3′) or 1099-1119 (5′-AAGATGCTGAACCATGCACCT-3′) [50], respectively.

### 4.4. GST Pull-Down Assay

GST pull-down analysis was performed to determine the interaction of hMSH4 with several lysine acetyltransferases. To generate recombinant proteins fused to glutathione *S*-transferase, the coding sequences of hMof and hGCN5 were cloned in-frame into the bacterial expression vector pGEX-6p-2 (Pharmacia, Piscataway, NJ, USA), whereas Flag-tagged hTip60 was cloned into pET-28a (Novagen, Madison, WI, USA). Bacterial expression of hMSH4 was generated from constructs hMSH4/pET-28a and hMSH4/pGEX-6p-2. Recombinant protein expression was carried out in BL21(DE3)-RIL cells (Stratagene, La Jolla, CA, USA). Glutathione-Sepharose 4B beads (Amersham Pharmacia Biotech, Piscataway, NJ, USA) were used to capture GST-fusion proteins from the soluble fractions of cell lysates. Captured proteins were analyzed by Western blotting.

### 4.5. Yeast Three-Hybrid Analysis

Yeast three-hybrid analysis of hMSH4, hMSH5, and HDAC3 interaction was performed as described previously [27]. Specifically, the HDAC3 coding sequence was cloned into pGADT7 (Clontech) to produce HDAC3-Gal4-AD fusion protein. The creation of pBridge (pB) based constructs, hMSH5/pB/hMSH4, hMSH4/pB/hMSH5, and GPS2/pGADT7 were described previously [27]. Positive protein-protein interactions were ascertained by the transcription activation of highly inducible GAL1 UAS driving *HIS3* and GAL2 UAS driving *ADE2* reporter genes in the host strain AH109, in which adenine and histidine prototrophy was monitored with SD/-Ade-Leu-His-Trp medium.

### 4.6. *In Vitro* Acetylation Assay

The *in vitro* analysis of protein acetylation was performed essentially based on the published experimental procedure [51]. Specifically, Myc-hMSH4 and Flag-hMof were expressed separately in 293T cells. Following validation of protein expression by Western blot analysis, immunoaffinity purifications by immunoprecipitation with α-Myc and α-Flag were performed. Immobilized proteins on Protein A/rProtein G-Agarose beads were washed twice with 1× PBS containing 0.1% Tween-20 (Sigma) (1× PBST), with a final wash in the acetylation reaction buffer (20 μM acetyl-CoA, 50 mM Tris-HCl, 0.1 mM EDTA, 1 mM DTT, and 10% glycerol, pH 8.0). Immobilized proteins were mixed and incubated in the fresh acetylation reaction buffer at 30 °C with constant agitation. Reactions were terminated by the addition of SDS loading dye after the removal of reaction buffer. Acetylated proteins were analyzed by Western blotting with the α-Acetylated-Lysine antibody.

### 4.7. Survival Analysis

RNAi of *C. elegans* was performed by the feeding method [52]. Wild type (N2) or *him-14(it44)* larvae were raised on *E. coli* transformed with empty vector (L4440) or *mys-2(RNAi)* at 20°C. When the worms reached L4 stage, they were exposed to IR (60 Gy) and allowed to recover for two hours. Individual worms were transferred daily to fresh empty vector (L4440) or *mys-2(RNAi)* plates. After transfer of the individual worms, embryos on each plate were counted. Three days later, live nematodes from the same plate were counted to calculate the hatching/survival rate.

### 4.8. NHEJ Reporter Assay

The NHEJ reporter analysis was performed as described previously [29]. Briefly, the 293T/#8-1 reporter cell line was transiently transfected with 4 μg pCBA-(I-*Sce*I) plasmid DNA by the use of Amaxa Nucleofector (Lonza Group Ltd, Allendale, NJ, USA). The appearance of GFP positive cells (relative NHEJ activity) was analyzed and recorded by FACS analysis of 10,000 to 25,000 cells (FACSCalibur, Becton Dickinson, Franklin Lakes, NJ, USA).

## 5. Conclusions

The acetyltransferase hMof and deacetylase HDAC3 coordinately regulate the levels of hMSH4 acetylation in response to DNA damage induced by IR.

## Figures and Tables

**Table 1:** Yeast three-hybrid analysis of hMSH4-hMSH5 and HDAC3 interaction.

	BD-fusion	“Native” HA-tagged	AD-fusion	His/Ade activation
1	BD		HDAC3	−
2	hMSH4	hMSH5	AD	−
3	hMSH5	hMSH4	AD	−
4	hMSH4		HDAC3	−
5	hMSH5		HDAC3	−
6	hMSH4	hMSH5	HDAC3	+++
7	hMSH5	hMSH4	HDAC3	−
8	hMSH4	hMSH5	GPS2	+++
9	hMSH5	hMSH4	GPS2	+++

## Abbreviations

DSB: DNA double strand break
IR: ionizing radiation
NHEJ: nonhomologous end joining
MMR: DNA mismatch repair
